# Ankle-targeted exosuit resistance increases paretic propulsion in people post-stroke

**DOI:** 10.1186/s12984-023-01204-w

**Published:** 2023-06-30

**Authors:** Krithika Swaminathan, Franchino Porciuncula, Sungwoo Park, Harini Kannan, Julien Erard, Nicholas Wendel, Teresa Baker, Terry D. Ellis, Louis N. Awad, Conor J. Walsh

**Affiliations:** 1 John A. Paulson School of Engineering and Applied Sciences, Harvard University, Boston, MA 02134 USA; 2 Sargent College of Health and Rehabilitation Sciences, Boston University, Boston, MA 02215 USA

**Keywords:** Resistive training, Gait biomechanics, Soft exosuit, Locomotor adaptation, Post-stroke rehabilitation

## Abstract

**Background:**

Individualized, targeted, and intense training is the hallmark of successful gait rehabilitation in people post-stroke. Specifically, increasing use of the impaired ankle to increase propulsion during the stance phase of gait has been linked to higher walking speeds and symmetry. Conventional progressive resistance training is one method used for individualized and intense rehabilitation, but often fails to target paretic ankle plantarflexion during walking. Wearable assistive robots have successfully *assisted* ankle-specific mechanisms to increase paretic propulsion in people post-stroke, suggesting their potential to provide targeted *resistance* to increase propulsion, but this application remains underexamined in this population. This work investigates the effects of targeted stance-phase plantarflexion resistance training with a soft ankle exosuit on propulsion mechanics in people post-stroke.

**Methods:**

We conducted this study in nine individuals with chronic stroke and tested the effects of three resistive force magnitudes on peak paretic propulsion, ankle torque, and ankle power while participants walked on a treadmill at their comfortable walking speeds. For each force magnitude, participants walked for 1 min while the exosuit was inactive, 2 min with active resistance, and 1 min with the exosuit inactive, in sequence. We evaluated changes in gait biomechanics during the active resistance and post-resistance sections relative to the initial inactive section.

**Results:**

Walking with active resistance increased paretic propulsion by more than the minimal detectable change of 0.8 %body weight at all tested force magnitudes, with an average increase of 1.29 ± 0.37 %body weight at the highest force magnitude. This improvement corresponded to changes of 0.13 ± 0.03 N m kg^− 1^ in peak biological ankle torque and 0.26 ± 0.04 W kg^− 1^ in peak biological ankle power. Upon removal of resistance, propulsion changes persisted for 30 seconds with an improvement of 1.49 ± 0.58 %body weight after the highest resistance level and without compensatory involvement of the unresisted joints or limb.

**Conclusions:**

Targeted exosuit-applied functional resistance of paretic ankle plantarflexors can elicit the latent propulsion reserve in people post-stroke. After-effects observed in propulsion highlight the potential for learning and restoration of propulsion mechanics. Thus, this exosuit-based resistive approach may offer new opportunities for individualized and progressive gait rehabilitation.

**Supplementary Information:**

The online version contains supplementary material available at 10.1186/s12984-023-01204-w.

## Background

Stroke is a leading cause of motor disability, with over 100 million survivors worldwide [1]. Of these survivors, over 80% are left with locomotor dysfunction [2], resulting in slow and asymmetric gait presentations [3]. The incidence of stroke is projected to continue increasing over the next few decades [4], and thus presents an imminent challenge for independence and quality of life [5] for members of our communities. Reduced propulsive force generated by the paretic, or more affected, limb is a major contributor to these impairments, and leads to the inability of the individual to effectively propel the body forward [6]. This reduced paretic propulsion is partially due to weakness in the paretic ankle plantarflexor muscles [7], which leads to reduced ankle torque production, a key driver of propulsion [8]. Consequently, there is growing interest in rehabilitation programs that aim to increase paretic propulsion by targeting ankle function during the stance phase, when forward propulsion is generated, towards achieving the functional outcome of increased gait speed [9].

Among methods that improve speed and propulsion, those that elicit the latent propulsion reserve through high-intensity training have been shown to be particularly promising [10]. The presence of latent propulsion reserve is typically demonstrated as an increase in an individual’s propulsion while increasing the difficulty of the locomotor task, such as by increasing surface inclination [11] or resisting the entire body during walking [12]. However, this extra propulsion can be generated by ankle-level mechanisms (i.e., ankle kinetics) or limb-level mechanisms (i.e., proximal kinematics) [8]. Simulations suggest that traditional methods of engaging the latent propulsion reserve, such as through passive resistive elements acting on the patient’s limbs (e.g., elastic bands attached at the pelvis opposing forward motion, or weights added to the foot) [12–14], typically result in larger involvement of the proximal joints and affect the entire gait cycle rather than targeting the ankle in stance [15]. Over the past two decades, several wearable robotic systems for assisting the ankle during walking have demonstrated the ability to increase paretic propulsion [16] through ankle-specific mechanisms [17]. Based on the principles of high-intensity and task-specific training [18], a wearable robotic system that *resists* the ankle during walking may be an important approach, particularly for patients with higher propulsive capacities. Currently, however, targeted resistance training of the paretic ankle plantarflexors during stance for people post-stroke has yet to be investigated.

Most robotic systems developed for resistance training emulate conventional methods [19–22], resulting in a lack of specificity to paretic propulsion. Wearable devices offer the capability to provide controlled torques to target a specific joint and phase in the gait cycle. Consequently, some groups have developed systems for targeted swing-phase resistance in post-stroke [23] and healthy populations [24, 25], and have shown adaptations in joint kinematics that indicate increased ankle use. More recent work has shown that targeted stance-phase resistance can increase plantarflexor muscle activity in people with cerebral palsy [26] and healthy individuals [27, 28]. However, people post-stroke present with gait biomechanics and adaptation responses to perturbations that are different from both of these populations [29–32]. Thus, there is a need to explore the use of a wearable resistive robotic system for increasing paretic propulsion in stance for people post-stroke.

One challenge for developing a resistive paradigm with a wearable device is identifying the appropriate parameters of resistance. Prior literature has shown the sensitivity of users to the magnitude of active ankle resistance in able-bodied individuals [28] and passive resistance in post-stroke individuals [33]. For example, excessive resistance can lead to compensatory gait patterns that increase use of the unresisted proximal joints or limb, as evidenced by changes in limb loading or joint kinematics. Perspectives from the challenge point theory [34, 35] further support the importance of individualizing the challenge level during training to maximize retention of the learned task. Thus, there is a need for structured investigation of the effects of resistance parameters on post-stroke gait response to stance-phase ankle resistance.

An effective resistance training paradigm is one that induces learning of increased ankle use towards generating propulsion. Evidence of learning in the motor learning field is often obtained from after-effects in the few steps immediately following a perturbation [25, 27, 36], representing the persistence of an individual’s adapted state [37]. However, measuring after-effects following exoskeleton-based training has traditionally been challenging due to the added distal inertia of rigid devices, which requires a user to first doff the device, and thus may prevent capturing newly learned gait patterns. By design, the cable-driven soft exosuit only consists of textile components at the distal end of the leg, and hence can be rapidly commanded to apply no forces by releasing tension in the cables (< 50ms) [38]. In this “slack” mode, the device is transparent to the user, resulting in similar kinematics and energetics to when walking without any device [39, 40]. This transparency allows for the measurement of gait immediately after resistance without stopping walking. This approach has been used to measure changes in ankle kinematics in healthy individuals after ankle-targeted resistance [28], but has yet to be applied to people post-stroke.

In this work, we leverage a soft, cable-driven, unilateral ankle exosuit [41] to investigate the biomechanical effects of targeted stance-phase ankle resistance across varying force magnitudes in chronic survivors of stroke. We hypothesized that with this targeted approach, we would engage individuals’ latent propulsion reserve through ankle-specific mechanisms, such as ankle kinetics and plantarflexor muscle activity. We expected to observe after-effects of increased propulsion compared to baseline for strides immediately following removal of the resistive force, due to the trained increase in ankle use. We also posited that with increased force magnitude, we would observe greater gains in propulsion metrics following resistance, but at the cost of increased use of the unresisted proximal joints and non-paretic limb, based on our prior work in healthy individuals [28]. To control for the effects of speed on joint kinetics and kinematics, we conducted this investigation on a treadmill with fixed walking speeds for each individual. We performed one additional proof-of-concept exploratory experiment to assess the value of an exosuit for resistive training in which individuals walked on a treadmill without any active resistance to quantify improvements in propulsion solely from treadmill training.

## Methods

The purpose of this study was to investigate how exosuit-applied stance-phase plantarflexion resistance affects propulsion and gait biomechanics in people post-stroke. The experiment was designed to measure joint kinetics and muscle activity during a range of exosuit resistance conditions.

### Participants

Nine individuals with chronic stroke (3 female; 114 ± 46 months post-stroke (mean ± std); age: 51.6 ± 9.0 years; mass: 78.4 ± 20.1 kg; height: 1.73 ± 0.09 m) were recruited to participate in this single-session study (Table 1). Seven participants had left-sided hemiparesis. All participants were naïve to exosuit resistance, except for one participant who was provided a substantial (6-month) washout period prior to the experimental visit. We recruited limited and full community ambulators [42] to ensure that individuals would be able to complete the protocol and walk for a duration of 30 min within a 3-hour session. All participants had a Functional Ambulatory Category score of 4 or higher [43–45]. All individuals provided medical clearance and written informed consent prior to participation. The study was approved by the Harvard Longwood Medical Area Institutional Review Board, and all methods were carried out in accordance with the approved study protocol.

**Table 1:** Participant Characteristics

Participant	Type of Stroke	Side of paresis	Sex	Age (yrs)	Chronicity (months)	Baseline Percent Propulsion*
1	Hemorrhagic	Right	M	37	95	35
2	Ischemic	Left	M	57	115	44
3	Hemorrhagic	Right	F	52	97	45
4	Hemorrhagic	Left	M	62	119	09
5	Ischemic	Left	F	52	85	31
6	Ischemic	Left	M	49	186	21
7	Ischemic	Left	M	60	82	59
8	Unknown	Left	M	38	190	39
9	Ischemic	Left	F	57	55	33

Participant baseline characteristics at the time of data collection. *Baseline percent propulsion represents the proportion of paretic propulsion impulse relative to the total propulsion impulse generated by both legs, such that 50 indicates perfect symmetry [9]. This metric has been used to classify impairment level in people post-stroke [46].

### Exosuit hardware and controller design

#### Hardware

**Fig. 1:**
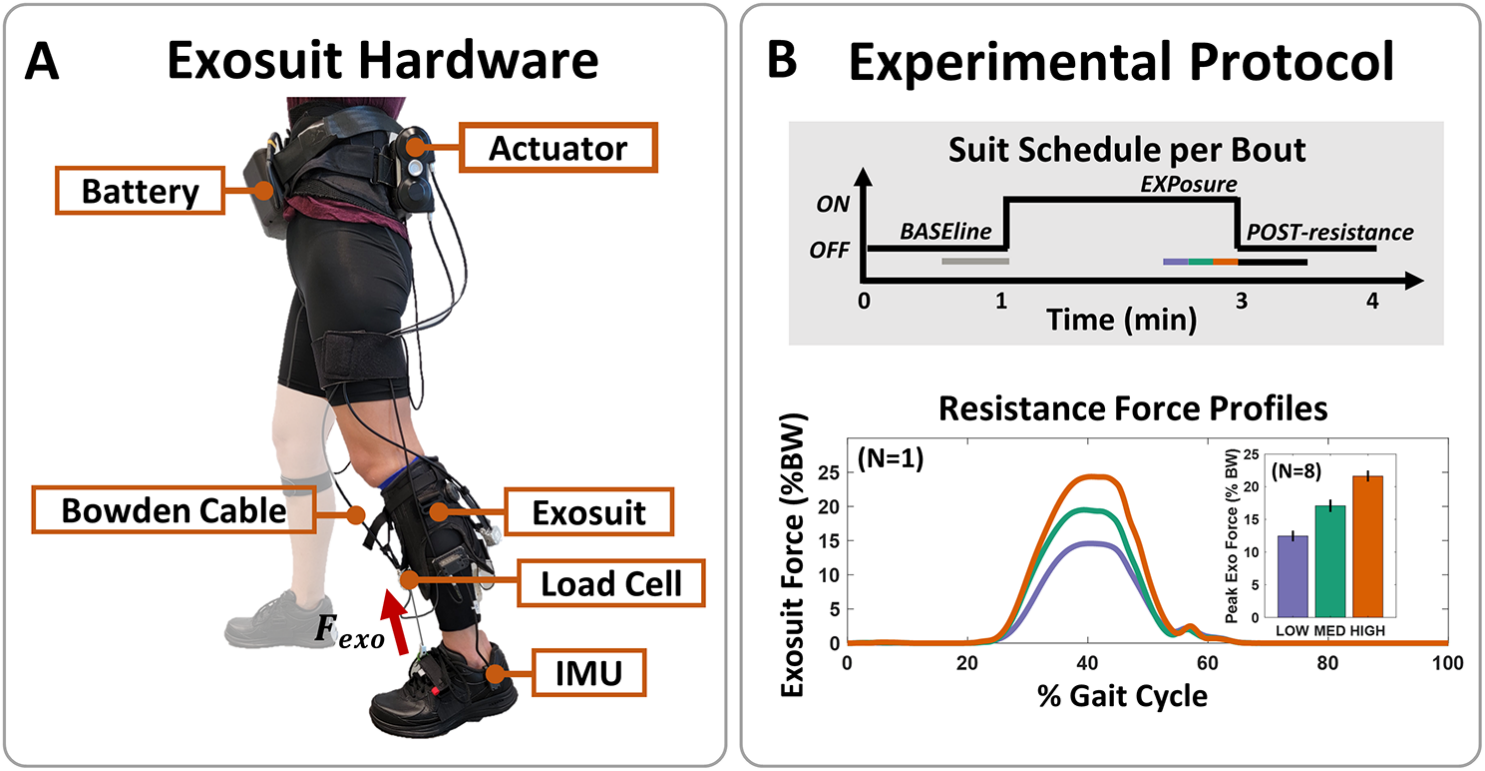
Exosuit hardware and experimental methods. (**A**) Soft ankle exosuit developed for post-stroke assistance and rehabilitation. **(B)** Overview of experimental protocol used in this study. (Top) Suit state (active/ON or slack/OFF) in each walking bout. (Bottom) Three force magnitudes were investigated. Example force profiles across the stride for a single participant and average peak applied force across all participants

For this study, we used the unilateral soft ankle exosuit previously developed by our team for assisting people post-stroke during gait (Fig. 1A) [41], and more recently modified for ankle plantarflexion resistance [28]. Briefly, the exosuit comprised an actuator unit mounted on a custom-designed waistbelt, Bowden cables that routed resistive forces from the actuator to the ankle, and a custom-designed calfwrap that provided attachment points for the cable and the body-worn sensors. The proximal anchor point of the cable was located on the anterior shin, while the distal anchor point was located on the dorsal midfoot area of the shoe. Torques were applied about the ankle by commanding a tension in the cable, which acted with a moment arm relative to the ankle joint center. A Fabrifoam® liner (Fabrifoam Products, Exton, PA, USA) was placed between the calfwrap and the calf to minimize drift and improve user comfort. Inertial measurement units (IMUs) were used to track the user’s gait cycle (GC), while a load cell was used to track the applied force in the cable (see Controller section for details). A battery unit located at the waist powered the system. The total weight of all exosuit components including the actuator and battery was 3.9 kg, with approximately 3.6 kg located proximally at the waist, and the remaining distributed along the length of the limb. We note that this exosuit also has a cable to assist ankle plantarflexion by design, but was disengaged throughout the duration of the study. Additional details on the original hardware design can be found in Bae et al. [41], and the weight breakdown by component is provided in Fig. S1.

#### Controller

When in the active mode, the load cell (LSB200, Futek, Irvine, CA, USA) measured the tension in the cable, and the IMUs (MTi-3, XSens, Enschede, Netherlands) were used to identify heel-strike and toe-off gait events for defining the force profile [41]. We used a multi-layered control strategy, building upon prior work from our group [47], in which the outer loop provided feedforward and feedback input based on the desired force, and the inner loop provided closed-loop control on the actuator motor velocity. The feedforward terms aimed to account for exosuit compliance while the feedback terms addressed error between the measured and commanded forces at each timestep.

The commanded force profiles were parameterized as piecewise functions, comprising two quintic splines that defined the rise and fall of the applied force. We set the timing of the force onset, peak, and offset, as well as the desired force magnitude at each point, to fully characterize the two spline functions. The onset force timing was set to 25% of the paretic limb’s single-support phase, close to the early onset times in a previous investigation of exosuit assistance profiles [48]. We targeted the earlier timing given prior evidence for increased after-effects [49]. The timing at which the peak force was applied was set to correspond to the participant’s natural peak ankle torque. This timing was obtained from an initial 2-minute treadmill collection during which subjects walked at their comfortable walking speed without any device. Similar ideas have been used in prior work in people with cerebral palsy [26] and unimpaired individuals [28]. While an adaptive control approach has been demonstrated in resistive exoskeletons for people with cerebral palsy by applying a resistive torque that is proportional to real-time estimated user biological torque, we chose to use a fixed controller to systematically investigate the effects of varying resistive force magnitude. The commanded peak resistance force magnitudes in our study were designed to correspond to 15, 20, and 25% of the participant’s body weight (BW), referred to as LOW, MED, and HIGH respectively (Fig. 1B). If the individual expressed discomfort at the HIGH condition, each force condition was shifted down by 5 %BW. These magnitudes were selected such that they were perceptible to participants without causing discomfort or exceeding actuator limits, and were identified through informal testing in a cohort of 4 post-stroke participants. The offset force timing was set such that the commanded force was 0 N by the start of the swing phase. When in the slack mode, the cable position was commanded to be constant throughout the entire stride such that the cable was not in tension. Thus, no forces were applied to the user (1.48 ± 0.20 N peak force across all analyzed slack sections for all subjects).

### Experimental protocol

Each participant walked on a treadmill at their comfortable walking speed (0.85 ± 0.23 m s^− 1^) while wearing the exosuit on their paretic limb for a series of 4-minute bouts. The comfortable walking speed was set prior to the start of the walking bouts by gradually increasing the treadmill speed in 0.05–0.1 m s^− 1^ increments until the participant found the speed too fast, and then decreasing back to the last comfortable speed to confirm. Participants were told that they would be asked to maintain this speed throughout the duration of the experiment without the aid of any assistive device (e.g., cane or ankle-foot orthosis). Each bout consisted of 1 min of slack walking, followed by 2 min of active resisted walking, and then by 1 min of slack walking performed continuously in sequence (Fig. 1B). For each bout, a researcher manually triggered the transition between the active and slack sections while the commanded force was 0 N, i.e., during the swing phase, to ensure participant safety and to prevent capturing a response to instability. The order of all force conditions (LOW, MED, HIGH) was randomized. We enforced rest breaks of 4 min between each bout, and individuals were allowed longer breaks if they reported fatigue. Participants were instructed to spend time on their paretic limb and push hard against the ground throughout the entire walking bout, both during the slack and active resistance periods. Specific instructions were provided given the known importance of task-specific instructions [50], and initial evidence that suggests explicit instructions may mitigate compensatory behavior in response to exosuit-applied resistance [28].

During the session, we collected optical motion capture data (Qualisys, Gothenburg, Sweden; 200 Hz) using a bilateral lower limb marker set. A total of 46 markers were placed across the two legs, with 6 per foot, 2 per ankle, 4 per shank, 2 per knee, 4 per thigh, and 4 per side of the pelvis. The final 2 markers were placed at the distal and proximal attachment points of the exosuit cable. We acquired three-dimensional ground reaction forces from an instrumented treadmill (Bertec, Columbus, OH, USA; 2000 Hz). Muscle activities from the soleus (SOL) and tibialis anterior (TA) were collected with surface electromyography (EMG) at 2000 Hz (Delsys, Natick, MA, USA). Exosuit sensor data from the on-board IMUs and load cell were streamed via Bluetooth at 100 Hz.

### Data analysis

#### Biomechanics

We first post-processed motion capture and ground reaction force data with a low-pass zero-phase filter with a 6 Hz cutoff to remove noise artifacts. Then, we used these filtered data to compute inverse dynamics with Visual3D biomechanics software (C-Motion, Germantown, MD, USA) and generate joint kinetics. Participant body mass was used to normalize all kinetic variables. Joint kinematics were also obtained through Visual3D.

For EMG data, we first applied a fourth-order Butterworth bandpass filter from 20 to 450 Hz. The data were then rectified and low-pass filtered at 6 Hz to get the signal envelope. Finally, for each subject and condition, we normalized the data by the average peak value across all strides in the last 30 s of the initial slack section. This approach aimed to reduce the effects of possible drift related to shifts in sensor location or changes in the skin-sensor interface throughout the duration of the experiment.

All data were segmented and interpolated to span from one heel-strike (0% GC) to the next, using force plate data to detect heel-strike events. Strides in which the participant crossed belts on the treadmill were removed from analysis. Conditions with less than 5 valid strides were excluded from statistical analysis. The number of subjects used for each biomechanical result is noted in the figures and text.

#### Suit

We calculated exosuit and biological contributions to net ankle joint kinetics, i.e., torque and power, by synchronizing and combining force data from the exosuit load cell with joint and cable kinematics data from motion capture as shown in prior work [51]. Briefly, markers placed on the distal and proximal cable attachments were used to compute the dynamic moment arm of the exosuit force relative to the ankle joint center throughout the gait cycle. Biological torque was computed by taking the difference between the net torque obtained from inverse dynamics and the resistive torque applied by the exosuit. Biological power was then obtained from the product of biological torque and ankle angular velocity.

#### Evaluation Metrics

We focused on propulsion metrics in this study, i.e., peak paretic propulsion, paretic propulsion impulse, and propulsion impulse symmetry. To evaluate whether our approach targets mechanisms specific to ankle plantarflexion, we investigated peak biological ankle torque and peak biological ankle power on the paretic limb. At the muscle-level, we calculated the average activity of the paretic soleus, a primary plantarflexor muscle, and the paretic tibialis anterior, a primary dorsiflexor muscle, during the stance phase, i.e., heel-strike to toe-off for each stride.

We also aimed to investigate compensatory behavior at the intra- and inter-limb levels. We first computed the average positive work done in the push-off phase by the proximal paretic joints, the knee and hip, during active resistance. This analysis was inspired by prior investigations of user biomechanical response to assistive exosuit forces in this population [17]. We then evaluated changes in stance-phase knee extension after resistance to assess whether paretic knee extensor activity increased since the applied plantarflexion resistance also transmits a knee flexor torque, and thus could be counteracted by either biological ankle plantarflexion or knee extension. We also investigated changes in paretic trailing limb angle, another correlate of propulsion [8, 52] that would indicate a shift away from an ankle-level strategy. Finally, we computed the vertical ground reaction force impulse during stance for the non-paretic limb as a measure of interlimb compensation, a compensation that was observed in unimpaired individuals in response to high resistive forces [28].

For each metric and condition, we evaluated changes in the participant’s gait during and immediately after exposure to the exosuit resistance relative to the corresponding baseline period. We used 30 s of data from each section, similar to prior work investigating propulsion modulation in people post-stroke [12, 53]. Specifically, the last 30 s of each bout’s initial slack section were used as the baseline (BASE), the last 30 s of the active section were used to evaluate user response during exposure to resistance (EXP for exposure), and the first 30 s of the post-active (POST) period were used to evaluate after-effects immediately after removal of resistance (Fig. 1B).

### Statistics

We used a separate linear mixed-effects model [54, 55] at each force condition to determine the effect of resistance on gait biomechanics during the different walking sections (BASE, EXP, and POST). For each metric of interest, the subjects were defined as the random-effects term in the model and the walking sections as the fixed-effects term. Specifically, BASE was used to define the reference level and the effects of EXP and POST were analyzed. We chose this design as linear mixed-effects models are able to avoid false positive problems arising from complex covariance relationships and from small sample sizes [56]. Importantly, the result of this model directly tests the effect of each walking section of interest (EXP and POST) with respect to a specified reference level (BASE) by examining the coefficients of the fixed-effects terms and their p-values. An alpha level of 0.05 was used to indicate significance. Residuals of the data were checked to satisfy normality assumptions of the model. The dependent variables for the linear mixed-effects models were the evaluation metrics defined in the earlier section (see Section Data Analysis: Evaluation Metrics for details). For all variables, we also compared baselines across experimental conditions to ensure that effects from preceding conditions were fully washed out by testing for a main effect of condition order.

Then, to investigate whether the force magnitude was associated with changes observed in propulsion metrics, we created another linear mixed-effects model using the applied force magnitudes as the fixed-effects term. The dependent variable in this model was the change in peak propulsion in POST from BASE.

One subject did not complete all of the conditions as the session was terminated early due to subject time constraints, and biomechanical data from some conditions were deemed unreliable due to loss of electrode contact or marker detachment. We report the final number of subjects used for each condition and variable alongside the statistical results in the corresponding figures and tables. All statistical analyses were run with custom MATLAB scripts (Mathworks, Natick, MA, USA).

### Exploratory study protocol

Since treadmill-based and goal-oriented training is known to be effective for increasing paretic propulsion [10], we conducted an additional exploratory study to investigate whether the exosuit resistance was more effective than extended practice on a treadmill. We invited two individuals from the main experiment (Subjects 4 and 6; 2 male; age: 55.5 ± 9.2 years, mass: 81.1 ± 6.9 kg, height: 1.76 ± 0.01 m) to return on a separate day for a treadmill-based training session. Individuals walked on the treadmill for a time-matched period, repeated bouts of 4-minutes each, at the speed selected in their first visit. The exosuit was worn during the experiment but remained in slack mode throughout. Following these slack bouts, a final 4-minute bout was conducted, in which the exosuit followed the same scheduling as in the main experiment to allow for within-day comparisons. The force condition that maximized peak propulsion after-effects in the first visit was applied during the active resistance period. The participants were instructed to focus on their paretic limb and push off hard against the ground as in the main experiment for all walking bouts. We collected the same measurements as in the main experiment and evaluated changes in peak propulsion. Descriptive statistics for basic features of the data (e.g., mean) were evaluated given the limited amount of data.

## Results

### Exosuit performance

Across all participants, the exosuit applied a peak force of 12.45 ± 0.86 %BW, 17.09 ± 1.01 %BW, and 21.62 ± 0.84 %BW in the LOW, MED, and HIGH conditions respectively, which correspond to absolute peak forces of 91.8 ± 11.13 N, 126.3 ± 14.5 N, and 167.0 ± 16.3 N. The RMSE of peak applied force across all participants and conditions was 0.68 %BW (equivalent to 5.2 N for the mean participant body weight), and was similar to tracking performance in prior exosuit work [28, 47, 57]. Across all participants, these forces resulted in peak resistive torques of 0.09 ± 0.01 N m kg^− 1^, 0.13 ± 0.01 N m kg^− 1^, and 0.16 ± 0.01 N m kg^− 1^ in the LOW, MED, and HIGH conditions respectively.

### Challenging the ankle with targeted exosuit resistance training to access paretic propulsion reserve

**Fig. 2:**
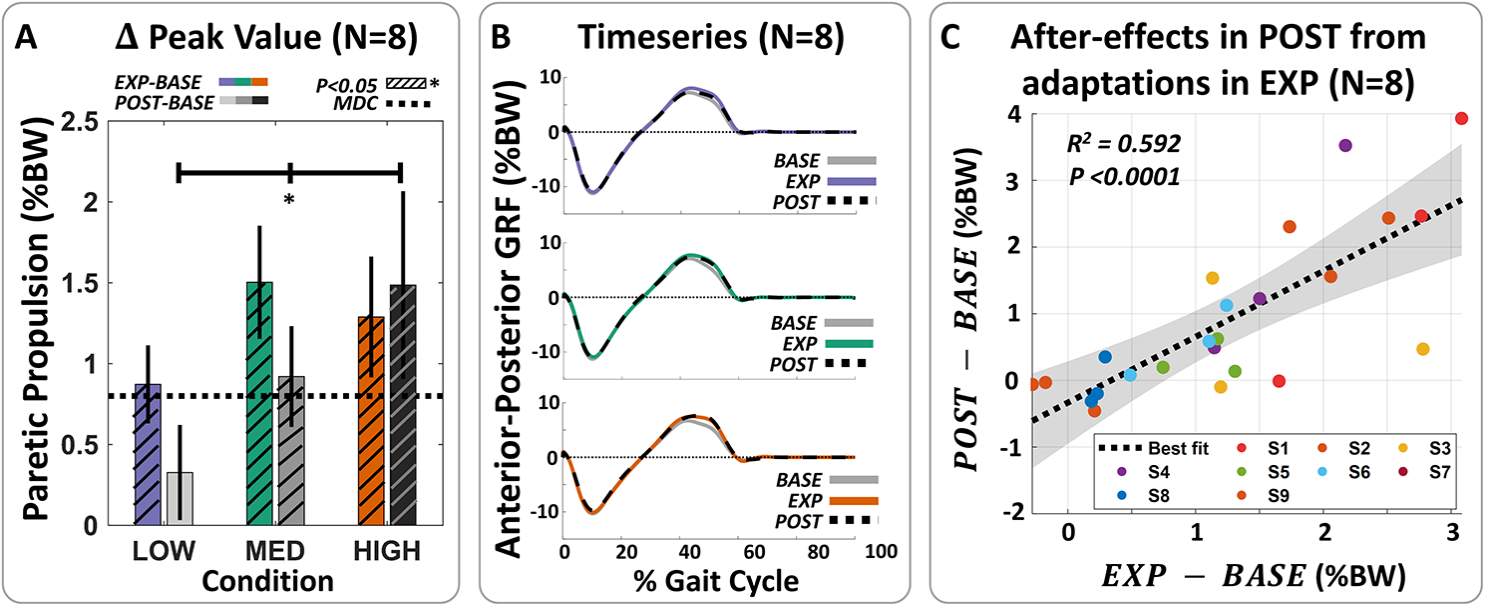
Propulsion increases during and after exosuit-applied resistance. (**A**) Difference between exposure and baseline (EXP-BASE) or post-exposure and baseline (POST-BASE) peak propulsion. Statistically significant increases relative to baseline (hatched bars) were observed in peak propulsion during and after exosuit-applied resistance. Increases surpassed the minimal detectable change (dashed line) [58] at the MED and HIGH resistive force magnitudes. A statistically significant effect of force magnitude (*) on after-effects (POST-BASE) was observed. (**B**) Average anterior-posterior ground reaction force (GRF) data across the gait cycle from all subjects during the baseline, exposure, and post-resistance periods. (**C**) Correlation between after-effects in peak propulsion during POST compared to BASE (y axis) and adaptations during EXP compared to BASE (x axis). A statistically significant moderate linear relationship was observed

**Fig. 3:**
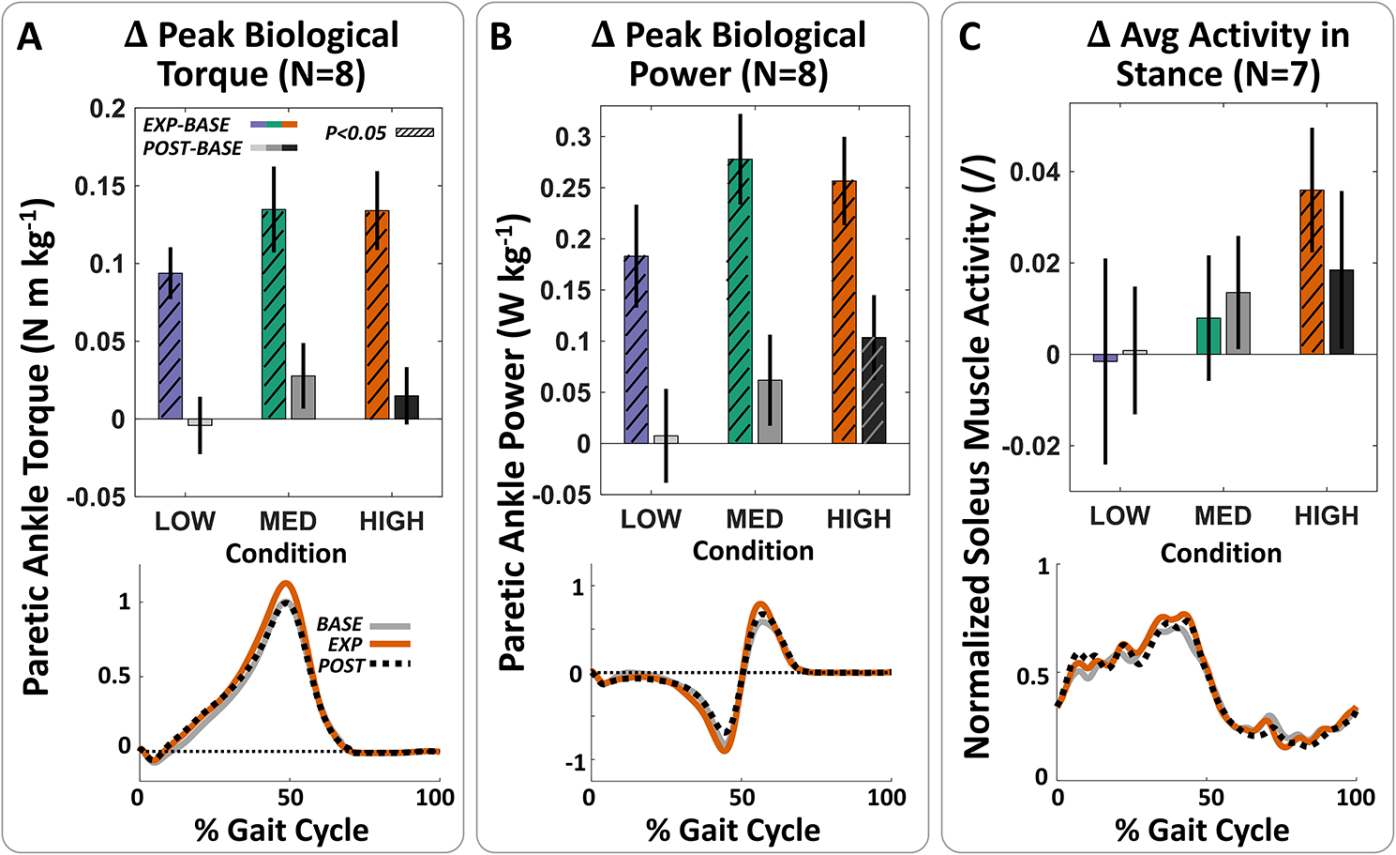
Plantarflexor torque, power, and muscle activity increase during exosuit-applied resistance. (**A**) (Top) Difference between exposure and baseline (EXP-BASE) or post-exposure and baseline (POST-BASE) peak paretic ankle biological torque. Statistically significant increases relative to baseline (hatched bars) were observed during exosuit-applied resistance at all resistive force magnitudes. (Bottom) Average biological ankle torque across the gait cycle from all subjects during the baseline, exposure, and post-resistance periods in the HIGH condition. (**B**) Paretic ankle biological power. A statistically significant increase in peak power was also observed after removing resistance in the HIGH condition. (**C**) Average normalized soleus activity during stance. Increased soleus activity was statistically significant only in the HIGH condition

**Fig. 4:**
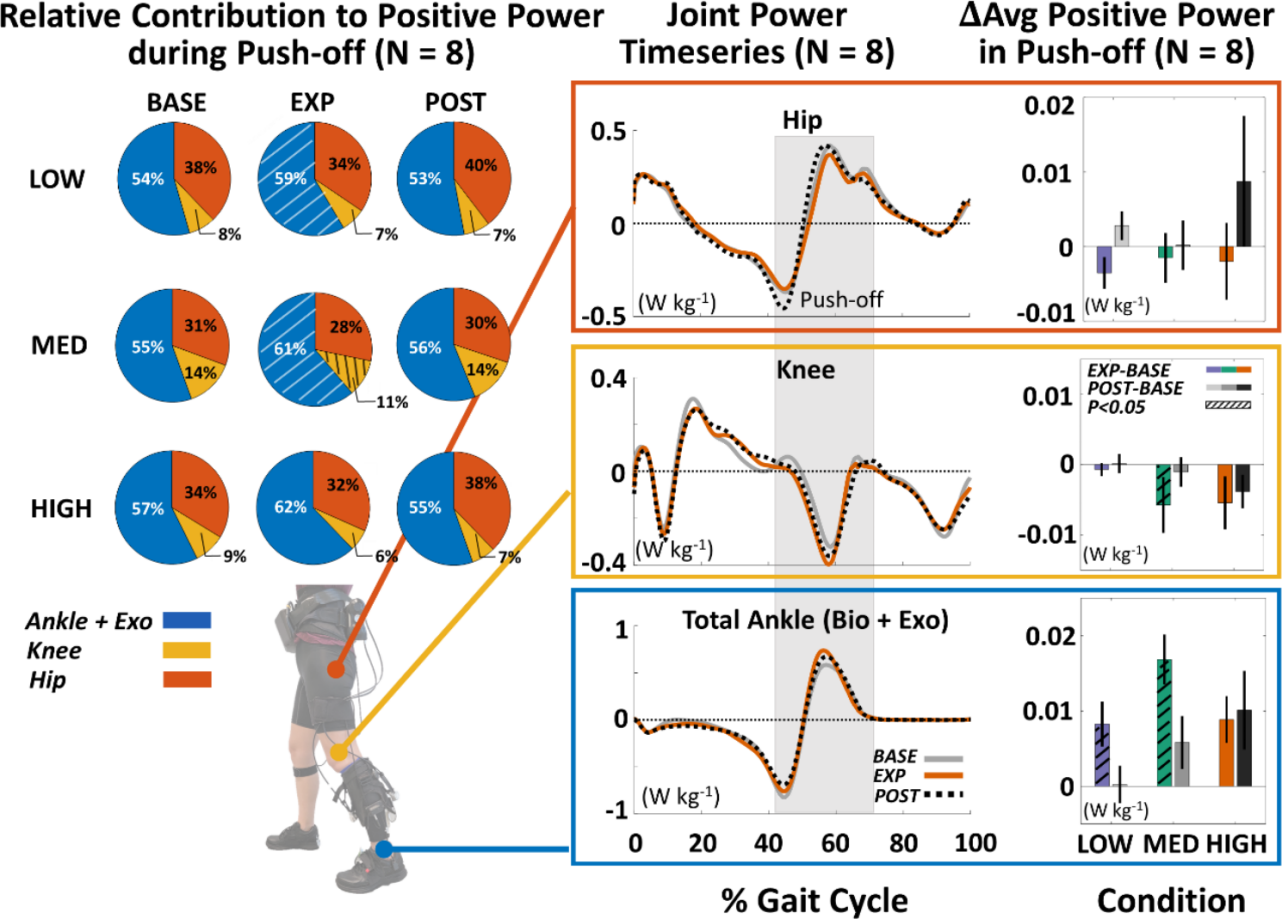
Propulsion reserve is accessed through increased ankle power in push-off. (Left) Group-level average contributions of the ankle, knee, and hip toward positive power during push-off. Using a similar approach to [17], we looked at the average positive power in the terminal double support phase across participants to compute their relative contributions. We find that net average ankle positive power increased significantly (p < 0.05) during the active resistance towards generating this additional propulsion. Statistically significant changes from baseline are indicated by hatched fill. **(Right)** Group**-**level timeseries joint power data for the HIGH force level and the average difference in positive joint power during push-off between exposure and baseline (EXP-BASE) or post-exposure and baseline (POST-BASE). Statistically significant increases (hatched bars) are observed at the ankle but not at the knee or hip joints

We found that across all force conditions, individuals significantly increased peak propulsion, propulsion impulse, and propulsion impulse symmetry during the active resistance periods (Fig. 2; Table 2). When comparing EXP to BASE, participants increased peak propulsion by 0.87 ± 0.24 %BW (p = 0.0020, n = 8) with LOW, 1.50 ± 0.35 %BW (p < 0.0001, n = 8) with MED, and 1.29 ± 0.37 %BW (p = 0.0028, n = 8) with HIGH resistance. These changes were complemented by significant increases in peak biological ankle torque and power (Fig. 3). At a group level, we observed an increase of 0.09 ± 0.02 N m kg^− 1^ (p < 0.0001, n = 8), 0.13 ± 0.03 N m kg^− 1^ (p < 0.0001, n = 8), and 0.13 ± 0.03 N m kg^− 1^ (p < 0.0001, n = 8) with LOW, MED, and HIGH forces, respectively. Peak biological power followed similar trends, with corresponding increases of 0.18 ± 0.05 W kg^− 1^ (p = 0.0016, n = 8), 0.28 ± 0.04 W kg^− 1^ (p < 0.0001, n = 8), and 0.26 ± 0.04 W kg^− 1^ (p < 0.0001, n = 8). We additionally found increases in average net ankle positive power during the terminal double support phase of stance, i.e., the push-off phase, that were significant in the LOW and MED force levels (p < 0.01, n = 8), but not in the HIGH condition (p = 0.0881, n = 8) (Fig. 4).

While we observed consistent increases in ankle kinetic variables, we found slight but significant group-level increases in soleus activity of 6.76 ± 2.72% only at the highest force magnitude (p = 0.0177, n = 7) (Fig. 3C). Conversely, we found decreases in average tibialis anterior muscle activity during stance, with reductions of -15.91 ± 3.98% (p = 0.0009, n = 6) and − 13.00 ± 2.67% (p = 0.0002, n = 6) in the LOW and MED conditions respectively, with no significant changes in the HIGH condition (p = 0.2086, n = 6) (Fig. S2). These results suggest that the combination of reduced dorsiflexor activity and increased plantarflexor activity collectively contributes to the observed increases in ankle kinetics, which in turn, increases propulsion.

**Table 2:** Changes in paretic propulsion metrics across force magnitudes relative to baseline

	Resistance Force Magnitude					
	**LOW**		**MED**		**HIGH**	
**Variable**	EXP	POST	EXP	POST	EXP	POST
Peak Propulsion (%BW)	**0.87 ± 0.24**	0.33 ± 0.29	**1.50 ± 0.35**	**0.92 ± 0.31**	**1.29 ± 0.37**	**1.49 ± 0.58**
Propulsion Impulse (%BW s)	**0.25 ± 0.08**	0.10 ± 0.06	**0.27 ± 0.10**	**0.20 ± 0.10**	**0.29 ± 0.12**	**0.34 ± 0.16**
Propulsion Impulse Symmetry (%)	**3.2 ± 1.6**	2.5 ± 1.5	**3.7 ± 1.8**	**3.2 ± 1.9**	3.5 ± 1.9	**5.0 ± 2.4**

Data are mean ± s.e.m. Each value is the difference relative to BASE for the corresponding condition. Statistically significant increases were observed in all measures of propulsion across the force levels investigated. N = 8 for all results presented. Bolded values indicate statistical significance (p < 0.05)

### After-effects of increased propulsion after removal of resistance

As hypothesized, we also observed significant increases in peak propulsion, propulsion impulse, and propulsion impulse symmetry in POST compared to BASE, with significant changes at the MED and HIGH force levels (Fig. 2; Table 2). Specifically, when comparing POST to BASE, participants increased peak propulsion by 0.33 ± 0.29 %BW (p = 0.1994, n = 8) after LOW, 0.92 ± 0.31 %BW (p = 0.0045, n = 8) after MED, and 1.49 ± 0.58 %BW (p = 0.0016, n = 8) after HIGH resistance. In parallel, average net ankle positive power during the push-off phase increased in the MED (p = 0.0558, n = 8) and HIGH conditions (p = 0.0632, n = 8). Peak biological ankle power increased significantly with HIGH resistance by 0.10 ± 0.04 W kg^− 1^ (p = 0.0094, n = 8) (Fig. 3B) as well, but changes in biological ankle torque and mean soleus activity were not significant.

We also found significant and positive moderate correlations between changes during the EXP period and changes in the POST period relative to BASE. Specifically, we found R^2^ values of 0.592 (p < 0.0001, n = 8), 0.510 (p < 0.0001, n = 8), and 0.228 (p = 0.0182, n = 8) for peak propulsion, biological ankle torque, and biological ankle power respectively, when using average data across all force magnitudes and participants (Fig. 2C). The corresponding regression slopes were 0.9872, 0.5495, and 0.4601. These relationships suggest that higher gains in propulsion and ankle-level kinetics achieved during the active resistance training period were associated with greater after-effects.

### Effect of resistance magnitude on learned increase of propulsion vs. compensatory mechanisms

**Fig. 5:**
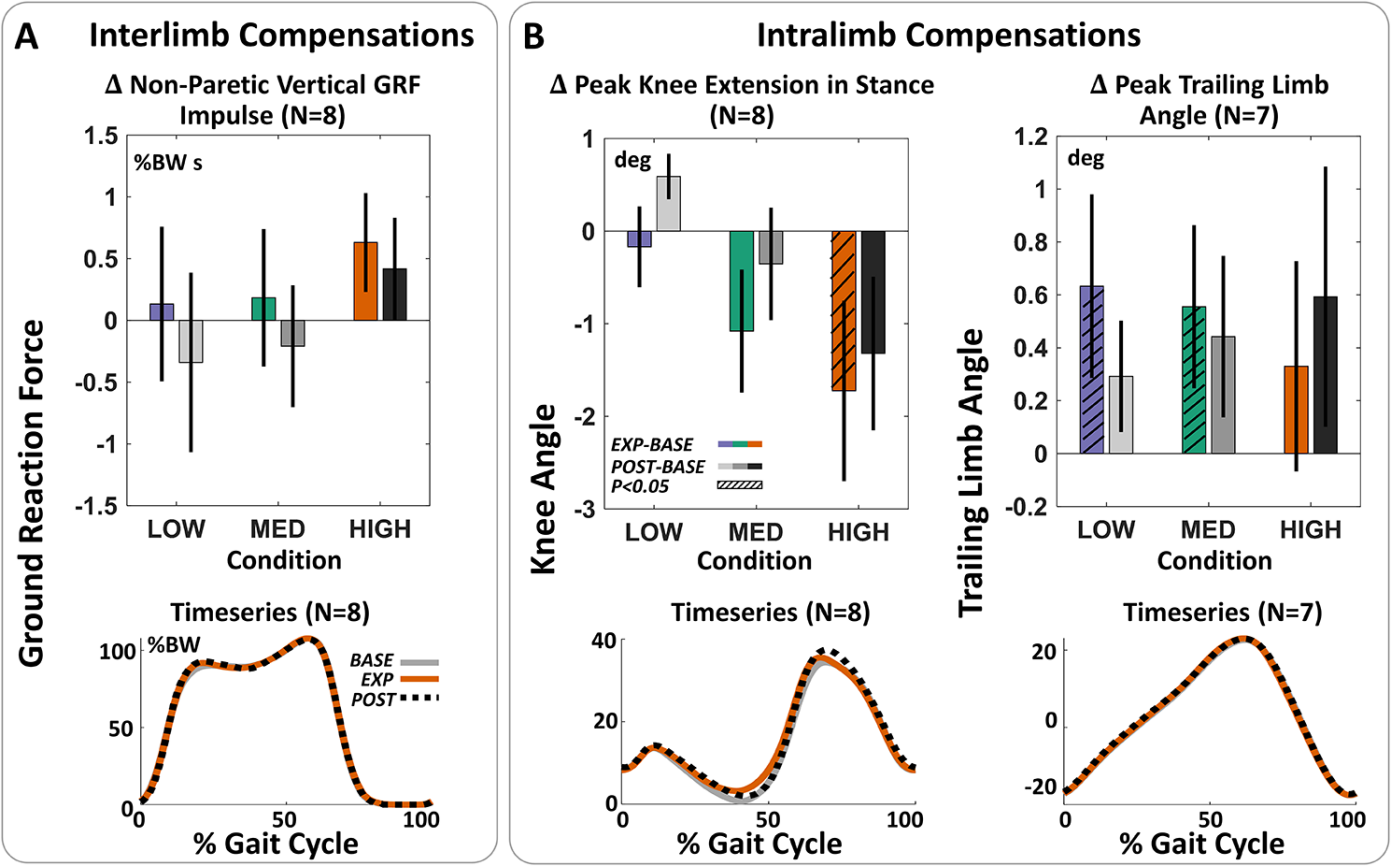
**No evidence of compensations at the non-paretic limb or unresisted proximal joints.** (**A**) Investigation of interlimb compensatory behavior. (Top) Average difference between exposure and baseline (EXP-BASE) or post-exposure and baseline (POST-BASE) non-paretic vertical ground reaction force impulse. (Bottom) Averaged non-paretic vertical ground reaction force across individuals in the HIGH condition. No statistically significant after-effects are observed at any force level. (**B**) Intralimb compensatory behavior. (Left) Paretic knee extension in early-stance through early swing (20–80% GC). Positive changes indicate increased knee extension. (Right) Peak trailing limb angle across the stride. Positive changes indicate increased trailing limb angle. No statistically significant after-effects are observed at any force level in either measure

We observed a significant main effect of force level (p = 0.0247, n = 8) on changes in peak propulsion in POST relative to BASE, which suggests that higher force levels may be associated with higher after-effects. Contrary to our hypothesis, we observed no significant changes in non-paretic limb loading, measured by the vertical ground reaction force impulse (p > 0.32, n = 8), even at higher force magnitudes (Fig. 5A).

At the intralimb level, maximum knee extension did not increase during stance in the POST period (< 0.6 deg, p > 0.07, n = 8), with an average reduction in extension in the HIGH condition (Fig. 5B, Fig. S3). Conversely, after HIGH resistance, we observed increased knee flexion during swing (p = 0.0011, n = 8) along with increased ankle dorsiflexion angle during stance (p = 0.0348, n = 8), suggesting improved tibial progression. Similarly, we did not observe any significant increases in peak trailing limb angle during the POST period at any force magnitude (p > 0.08, n = 8). Thus, in our sample, for the range of forces we investigated, we observed increases in ankle effort without compromising overall gait quality.

### Role of the exosuit-applied resistance in increasing propulsion

In the exploratory experiment, both participants exhibited less improvements in propulsion with the treadmill alone compared to with the exosuit. Averaged across all treadmill-only walking bouts, Subject 4 increased peak propulsion by 0.02 ± 0.25 %BW during the times corresponding to the EXP period (2.5-3 min) and reduced by 0.40 ± 0.19 %BW in the times corresponding to the POST period (3-3.5 min). In contrast, this participant increased peak propulsion by 1.84 ± 0.26 %BW during EXP and by 0.88 ± 0.38 %BW during POST in the active exosuit bout (Fig. S4, Data S3). A similar trend was seen in Subject 6, who increased peak propulsion in the treadmill-only bouts by 0.26 ± 0.18 %BW and reduced by 0.03 ± 0.19 %BW relative to BASE in the times corresponding to EXP and POST, respectively. In the active exosuit bout, peak propulsion increased by 1.87 ± 0.31 %BW and 0.82 ± 0.27 %BW in EXP and POST, respectively. Thus, the exosuit enabled approximately 7-92x more improvement in propulsion during the training period compared to a treadmill alone. These results, while only with two participants, suggest the importance of the exosuit for eliciting the observed propulsion improvements.

## Discussion

In this work, we have shown the feasibility and efficacy of a soft exosuit applying targeted ankle resistance for functional resistance gait training in people post-stroke. We found that individuals significantly and clinically meaningfully increased their peak paretic propulsion during active resistance. Moreover, the changes in gait during active resistance corresponded with the after-effects seen during the post-resistance period, suggesting that adaptations to the applied forces persisted after resistance was removed. We additionally observed increases in paretic ankle power during and after exosuit-applied resistance corresponding to changes in propulsion, suggesting that propulsion increases were generated through ankle-specific mechanisms. This study further showed that resistance force magnitude is an important factor for determining the size of after-effects. Finally, we demonstrated the potential benefit of exosuit-based resistive training over standard treadmill-based training. Altogether, these preliminary findings demonstrate the viability of exosuit resistance for further enhancing progressive gait rehabilitation for hemiparetic walking.

This technology and approach offer the opportunity for targeted resistance training that engages specific mechanisms that are relevant to the task. Indeed, the use of robotics for providing targeted resistance has demonstrated benefits in other populations such as in elderly individuals [59] and people with cerebral palsy [26]. Resistance training during stance is particularly relevant for less impaired individuals post-stroke who have residual propulsion reserve, such as those who participated in this study. An active system can additionally be tailored to an individual’s gait pattern and impairment level. Here, we showed that the force magnitude influences how users adapt to stance-phase resistive forces, suggesting that this tool may be used for modulating resistance parameters as users gain strength and function. Generalizing further, the exosuit is also capable of assisting people post-stroke by augmenting paretic ankle torque [48] and thus may enable individualizing training from assisted, to assist-as-needed, to resisted, a common progression seen in clinical rehabilitation [60].

By increasing the challenge level during training, we aimed to access the latent propulsion reserve in our participants through ankle-specific mechanisms. We found that when actively resisted by the exosuit, participants achieved meaningful improvements in peak propulsion and propulsion impulse based on corresponding minimal detectable change (MDC) thresholds, which are of clinical relevance [58]. Propulsion impulse symmetry also improved significantly and was close to the MDC level of 3.92% with MED resistance (3.7%). This increase during active resistance indicates that these participants had sufficient reserve to generate more propulsion than what was required to exactly offset the applied resistance. For context, the magnitude of effects in the current study are in line with other studies that sought to access propulsion reserve by applying a passive constant force at the waist [12]. We found that changes in peak propulsion with the exosuit-applied resistance are similar in magnitude to those seen when applying a passive force of 2.5% BW, and changes in propulsion impulse symmetry are similar to applying between 5 and 7.5% BW of passive force. However, while simulations of constant resistance at the pelvis suggest that there are minimal changes in ankle torque and power [15], we observed significant increases in biological ankle torque and power with active exosuit resistance. We note that this is not an expected result as individuals have highly heterogeneous responses to wearable devices for gait and can achieve the necessary power to maintain a given walking speed through numerous mechanisms. In particular, prior work has shown that increasing task difficulty may lead to a redistribution of power generation across the limbs and joints away from, rather than targeting, the impaired ankle [61, 62]. However, our participants did not exhibit increases in average knee or hip positive power during push-off at any force magnitude, which further supports the specificity of this approach within the range of resistance tested. Together, these results suggest that a joint-targeting exosuit may be able to better engage the paretic ankle, rather than limb-level mechanisms, to tap into the individual’s propulsion reserve.

Despite significant increases in ankle torque and power, we did not observe significant increases in plantarflexor muscle activity across force magnitudes. We posit that this may be due to a combination of factors relating to the muscle’s passive and active dynamics. First, by applying a resistive torque to oppose the plantarflexors, we may also be aiding with dorsiflexion angle in mid-stance. The resultant added tibial progression may lead to more effective passive muscle-tendon energetic exchange. Specifically, as muscle economy depends on the muscle’s length and velocity, by applying resistance relatively early in the gait cycle, we may be increasing isometric force generation, which is highly economical (more force generation per unit muscle activation [63, 64]). Moreover, the soleus is one of three muscles within the triceps surae. Thus, reductions in soleus activity may have been coupled with compensations in the other plantarflexors to maintain overall plantarflexion torque [65]. However, given that the other major plantarflexors, the gastrocnemii, are bi-articulate and are affected by both ankle and knee kinematics, this experiment did not evaluate changes in these muscles. Finally, the increased ankle torque may also be partially explained by increased reciprocal inhibition between the ankle plantarflexors and dorsiflexors. That is, smaller increases in the soleus muscle in conjunction with reductions in the antagonist muscle, the tibialis anterior, may result in larger changes at the joint level. Consistent with this hypothesis, we observed large significant reductions in tibialis anterior activity in our participants. Thus, future work may investigate changes in both muscle-tendon dynamics and reciprocal inhibition in the presence of targeted ankle resistance to focus on training specific muscle-level mechanisms towards generating propulsion.

Beyond accessing participants’ propulsion reserve during training, we aimed to capture evidence of learning by measuring after-effects in propulsion immediately after removal of exosuit-applied resistance. In this study, we use an analogous neuromotor learning concept to augmenting the paretic ankle deficit [66], to induce increased ankle use towards increased propulsion. Quantitatively, we found that changes in propulsion metrics during the post-resistance period exceeded the MDC thresholds. Qualitatively, participants reported improved ability to perceive and focus on their impairment with the applied resistance. The magnitude of these after-effects were also consistent with those observed by Lewek et al. with whole-body resistance [12]. These results suggest that exosuit resistance is able to generate clinically meaningful after-effects that last for 30 s after just two minutes of exposure to the training stimulus despite only targeting the paretic ankle. Future intervention-based trials with exosuit resistance are needed to validate the relationship between immediate after-effects and long-term learning mechanisms.

This study also aimed to investigate the effects of resistance magnitudes on gait biomechanics during and after resistance given the known dependence of user response to exoskeleton and exosuit parameters [67]. Based on the challenge point theory [34], we expected that the intermediate force magnitude would best target the paretic ankle. Instead, we observed that propulsion after-effects continued to increase with increasing resistance force magnitudes for the range of forces we tested. This finding corroborates past work that has demonstrated that increasing intensity is associated with increased motor learning [68]. Surprisingly, we found little evidence of compensatory behavior in response to the HIGH applied forces in this study during or after active resistance. This contrasts with prior work using passive resistive orthoses in which increasing resistance led to reductions in peak net and biological ankle power during resistance [33]. Thus, by specifically targeting the mid-late stance phase during which propulsion is generated, we may be able to avoid compromising ankle work across the stride. These findings also contrast with our prior work in unimpaired individuals [28] in which different magnitudes of stance-phase resistance were similarly applied unilaterally at the ankle. One reason for this difference may be that we did not apply forces that post-stroke participants deemed intolerable, and thus we may not have reached the paretic ankle’s maximum capacity for generating force. We additionally provided explicit instructions for participants to “push off against the ground” and “spend time on their paretic side,” which may have encouraged gait patterns that leveraged the paretic ankle rather than the unresisted joints. The importance of instructions to prevent compensatory gait patterns has been reported in similar targeted robotic resistance paradigms in unimpaired populations [28, 69]. Thus, we posit that the combination of well-parameterized forces and goal-oriented instructions allowed for functional resistance training with a wide range of resistance forces. Future work may investigate higher force magnitudes or longer timescales of training to understand whether compensatory behaviors emerge when the ankle is at capacity or fatigued.

While this work showed promising results, we note that there are a few limitations that future work should investigate further. First, participants chosen for this study were generally less impaired, with an average speed of over 0.85 m s^− 1^, as individuals needed sufficient endurance to complete the experiment. Nevertheless, these individuals presented with propulsion deficits even in their chronic stage of recovery. Interestingly, we observed similar magnitudes of improvement in propulsion during active resistance to active assistance applied by the same device in a previous study [48], but we did not conduct a direct comparison between the two training methods in this work. Thus, there is a need to identify which individuals are more likely to benefit from assistance versus resistance based on their impairment levels and propulsion reserve. Indeed, the improved efficacy of resistance training in less impaired individuals has been observed in whole-body robotic rehabilitation [70]. Second, while in this study we provided specific instructions to oppose the exosuit resistance to mimic a potential clinical implementation, we acknowledge that the ability of exosuit-applied resistance to generate propulsion changes without any instructions remains unexplored in people post-stroke. This study also was conducted with a fixed controller at fixed speeds to assess the experimental variables but given the dependence of gait biomechanics on walking speed, future work should investigate the use of adaptive controllers at varying speeds to maximize training effects. Finally, we note that while we focused on resistive force magnitudes, further investigation is needed on other resistance parameters such as timings.

## Conclusion

In summary, through this work, we aimed to investigate the hypothesis that targeted plantarflexion resistance training with an exosuit would increase paretic propulsion through ankle-specific mechanisms in people post-stroke. We further studied the effects of systematically varying the resistance magnitude on gait biomechanics. As hypothesized, we observed significant and clinically meaningful improvements in paretic propulsion and paretic ankle kinetics during active resistance at all resistance levels. We also observed after-effects in paretic propulsion, with higher resistance during training leading to larger after-effects but without evidence of compensatory behavior. This study represents an important step in wearable rehabilitation robotics for retraining post-stroke propulsion through improved ankle mechanics that extends beyond providing immediate assistance to generating motor adaptations. Future work may leverage these results to further optimize rehabilitation protocols for individuals of varying impairment levels, and to capitalize on latent propulsion reserve in this community.

## Electronic supplementary material

Below is the link to the electronic supplementary material.

Additional File 1: Hardware component breakdown

Additional File 2: Stance-phase tibialis anterior activity across force magnitudes

Additional File 3: Joint kinematics across force magnitudes

Additional File 4: Additional study: Effect of the exosuit vs treadmill walking

Additional File 5: Experimental data for the paper’s main and exploratory studies

## Data Availability

All data generated or analyzed during this study are included in this published article and its Supplementary Information files.

## Abbreviations

BASE: Baseline
BW: Body Weight
EMG: Electromyography
EXP: Exposure to resistance
GC: Gait cycle
GRF: Ground Reaction Force
IMU: Inertial measurement unit
MDC: Minimal detectable change
POST: Post-exposure to resistance
SEM: Standard error of the mean
SOL: Soleus
TA: Tibialis Anterior
