# Supplementary figures and images for: Ankle-targeted exosuit resistance increases paretic propulsion in people post-stroke
Source: J Neuroeng Rehabil. 2023 Jun 30;20:85. doi: 10.1186/s12984-023-01204-w (PMC10314463; doi:10.1186/s12984-023-01204-w)

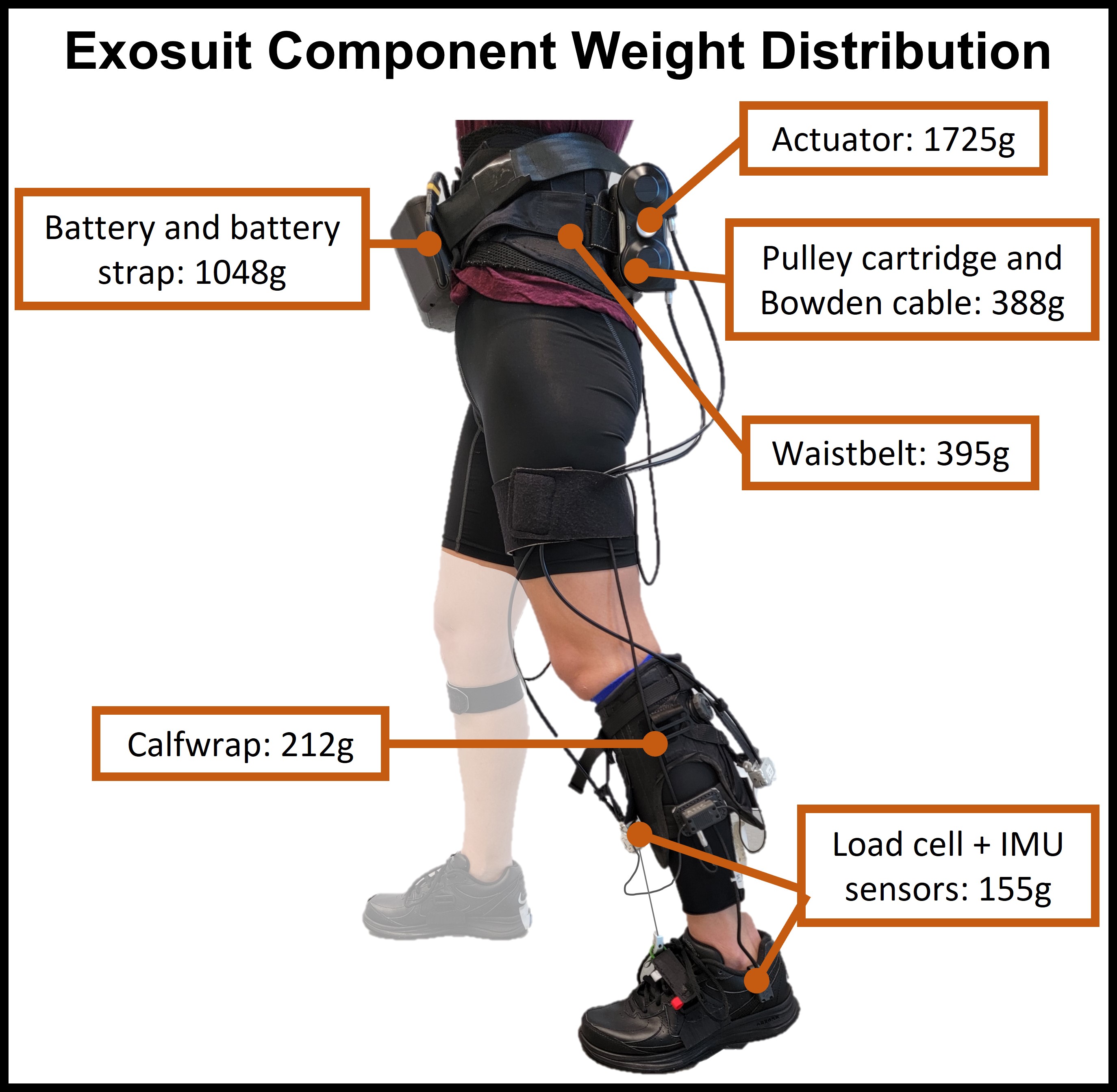

Supplement: Supplementary file 1 — Additional File 1: Hardware component breakdown [file 12984_2023_1204_MOESM1_ESM.jpg]

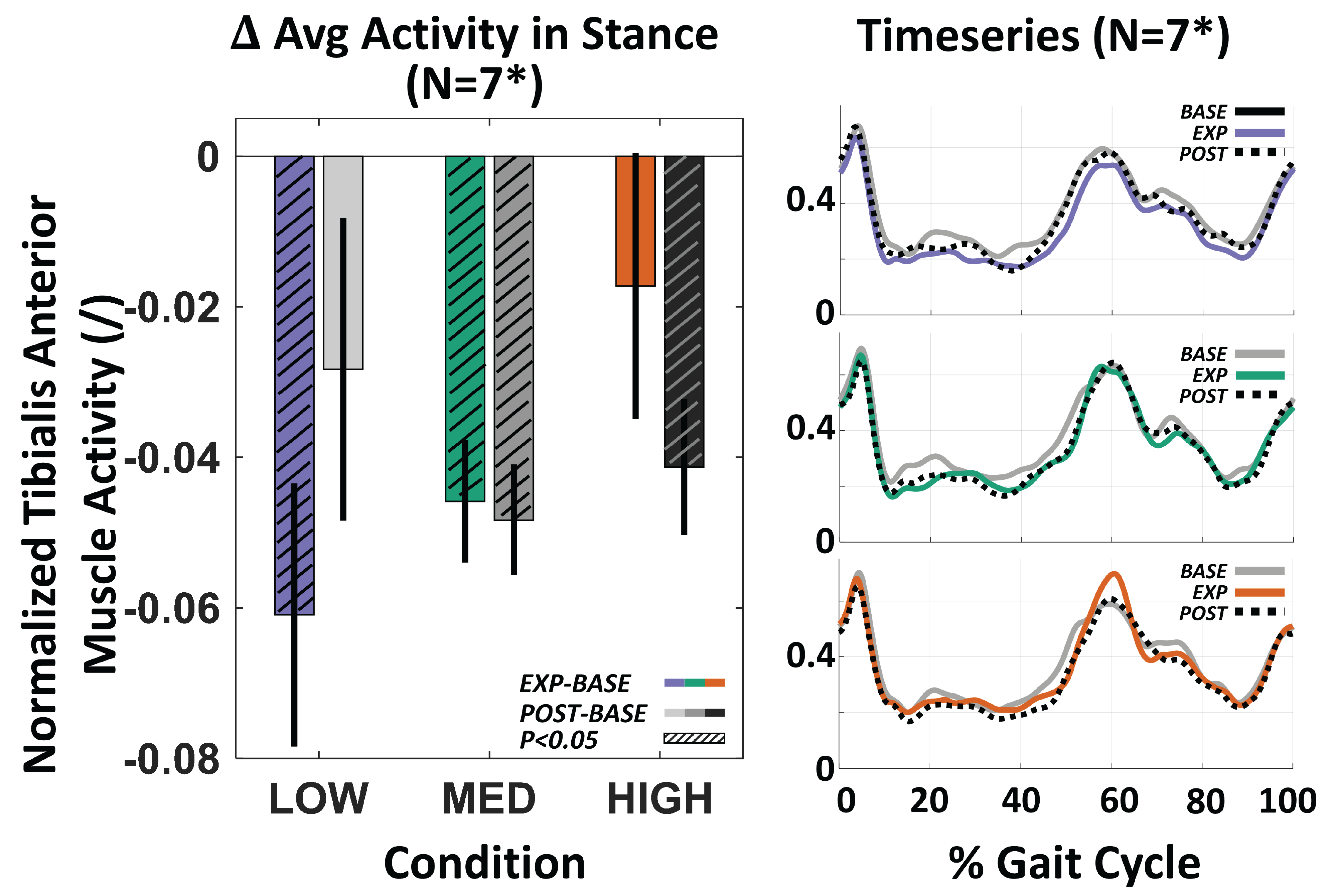

Supplement: Supplementary file 2 — Additional File 2: Stance-phase tibialis anterior activity across force magnitudes [file 12984_2023_1204_MOESM2_ESM.png]

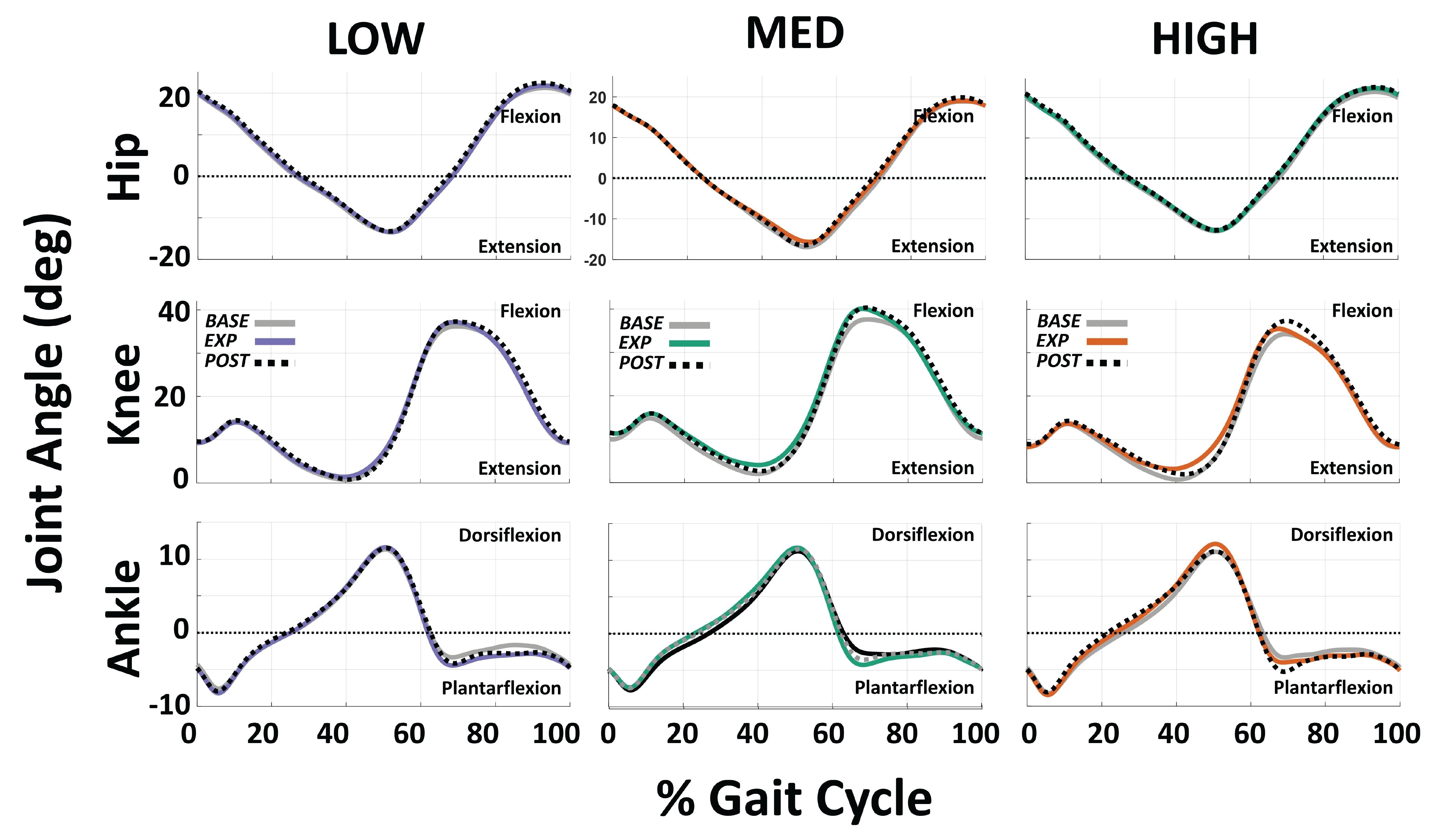

Supplement: Supplementary file 3 — Additional File 3: Joint kinematics across force magnitudes [file 12984_2023_1204_MOESM3_ESM.png]

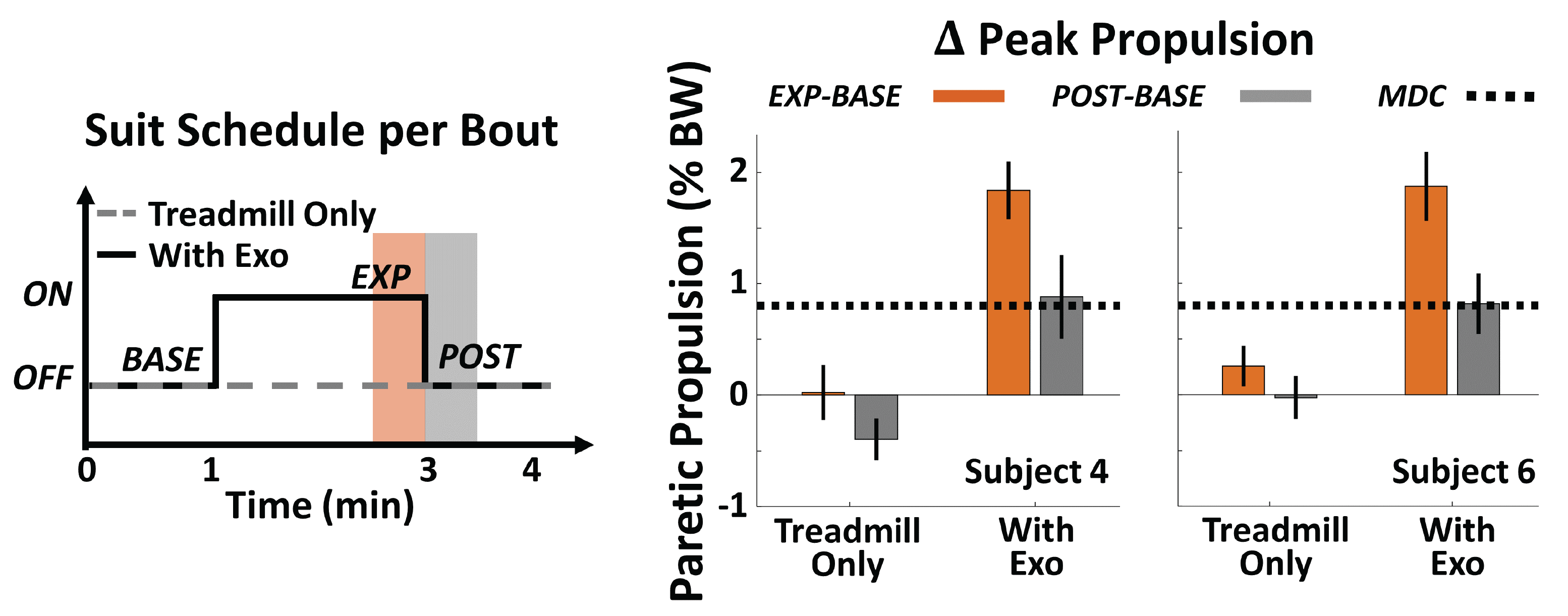

Supplement: Supplementary file 4 — Additional File 4: Additional study: Effect of the exosuit vs treadmill walking [file 12984_2023_1204_MOESM4_ESM.png]
